# Subcapsular Liver Hematoma: A Rare Complication of Hemolysis, Elevated Liver Enzymes, and Low Platelets (HELLP) Syndrome Managed Conservatively

**DOI:** 10.7759/cureus.22058

**Published:** 2022-02-09

**Authors:** Stylianos Grigorakis, George N Tzimas, Chalent Alexakis, Beatrice E Morea, Nikolaos Kontomitros

**Affiliations:** 1 Obstetrics and Gynaecology, Iaso Maternity Hospital, Athens, GRC; 2 Hepatobiliary Surgery, Hygeia Hospital, Athens, GRC; 3 Obstetrics and Gynaecology, Carol Davila University of Medicine and Pharmacy, Athens, GRC; 4 Obstetrics and Gynaecology, Carol Davila University of Medicine and Pharmacy, Colorado, USA

**Keywords:** subcapsular hepatic hematoma, gestational hypertension, liver disease of pregnancy, emergency cesarean section, hellp syndrome

## Abstract

HELLP syndrome is an acronym used, since 1982, to describe a combined disorder of the liver and coagulation cascade defined as pre-eclampsia in pregnant women with hemolytic anemia, an increase in liver enzymes, and a decrease in platelet count. Spontaneous liver rupture is an exceptionally rare and extremely severe, occasionally lethal, complication of pre-eclampsia - eclampsia and especially hemolysis, elevated liver enzymes, low platelets (HELLP) syndrome. The following report describes a case of a 48-year-old woman diagnosed with HELLP syndrome complicated by spontaneous liver rupture who was treated conservatively.

## Introduction

Spontaneous liver rupture (SLR) is a rare life-threatening complication of preeclampsia - eclampsia and of HELLP (hemolysis, elevated liver enzymes, low platelets) syndrome. Although clinical presentation is with nonspecific pain (epigastric, right upper quadrant, and shoulder), it should, however, provide a high index of suspicion. The diagnosis of subcapsular liver hematoma (SLH) must lead to urgent delivery through cesarean section. The incidence of SLH rupture in pregnancy varies from 1/40,000 to 1/250,000 and has been reported in less than 2% of pregnancies complicated by HELLP syndrome [1]. In this frame, a case pertaining to a 48-year-old woman with a grade III SLH secondary to HELLP syndrome, which was non-operatively managed, is presented.

## Case presentation

This case describes a 48-year-old gravida 0, para 0 female of Indian origin who underwent in-vitro-fertilization (IVF) pregnancy with egg donation and was diagnosed with pre-eclampsia at 33 weeks (elevated blood pressure and proteinuria of 1+ on urinary dipstick test). The patient presented at 35+3 weeks of gestation in the maternity hospital for evaluation where an elevated uric acid was noted (8.3 mg/dL). Corticosteroids were administered and a cesarean section was scheduled after two days. At 35 weeks and four days of gestation, the patient presented in the hospital with severe epigastric pain, right upper quadrant pain, shoulder pain, vomiting, and increased blood pressure (BP) noting: 150/90 mmHg. Past medical and family history was unremarkable. Vital signs on admission were all within normal limits except an elevated BP 150/90 mmHg and uric acid 9.2 mg/dL (normal range: 2.6-6.2 mg/dL). Initial laboratory workup before the cesarean section showed the following; hematocrit (HCt): 39.2% (N: 36%-45.5%), hemoglobin (Hb): 12.7 g/dL (N: 12-15.8 g/dL), platelet count (PLT) 187,000/μL (N: 150-450 × 10^3^/μL), white blood cell count (WBC): 12,620 cells/mm^3^ (N: 4.5-11.0 × 10^3^/μL), aspartate aminotransferase (AST): 54 IU/L (N: 15-37 IU/L), alanine aminotransferase (ALT): 53 U/L (N: 14-59 IU/L), lactate dehydrogenase (LDH): 326 IU/L (N: 81-234), alkaline phosphatase 161 IU/L (N: 46-116 IU/L), creatinine 0.97 mg/dL (N: 0.5-1.1 mg/dL), and urea 48 mg/dL (N: 15-39 mg/dL). Coagulation profile included examination of partial thromboplastin time (PTT) and activated PTT (aPTT), which were within normal ranges but fibrinogen was found 445 mg/dL (N: 180-350 mg/dL) and thrombin time (TT) 28.9 sec (N: < 21 sec) (Table 1).

**Table 1:** Dynamic evolution of the laboratory exams before C-section and for the following three days post-operatively. (PT = Prothrombin Time, INR = International Normalized Ratio, ALT = Alanine Aminotransferase, AST = Aspartate Aminotransferase, LDH = Lactate Dehydrogenase)

Laboratory exam	Before C-section (15/02, 21:00)	16/02, 02:00	16/02, 16:00	16/02, 18:00	17/02, 03:00	17/02, 18:00	18/02, 04:00
Leukocytes (4.5–11.0 × 10^3^/μL)	12.62	14.37	20.56	19.05	13.77	9.71	14.48
Hemoglobin (12.0–15.8 g/dL)	12.7	12.1	12.5	9.2	8.4	9.2	8.2
Hematocrit (36.0–45.5%)	39.2	36.5	37.5	28.3	25.6	27	24.2
Platelets (150–450 × 10^3^/μL)	187	121	104	120	~69	~60	~54
PT (9.8–11.7 Seconds)	11.3	11.3	11.7	11.3	12.1	15	17.7
INR (0.9–1.1)	1	1	1	1.4	1.4	1.4	1.5
Thromboplastin time (<21 sec)	28.9	37.1	23.1	24.8	35.1	24.1	19
Fibrinogen (180–400 mg/dL)	445	368	413	300	355	368	377
Creatinine (0.5–1.1 mg/dL)	0.97	0.85	0.99	1.3	1.21	1.31	1.34
Uric Acid (2.6–6.2 mg/dL)	9.2	7.8	-	-	8.6	-	9.6
ALT (14–59 Unit/L)	53	631	520	393	2,743	4,852	4,874
AST (15–37 Unit/L)	54	799	383	295	2,683	3,650	5,056
LDH (81–234 Unit/L)	326	795	673	538	2,180	-	2,326

**Figure 1:**
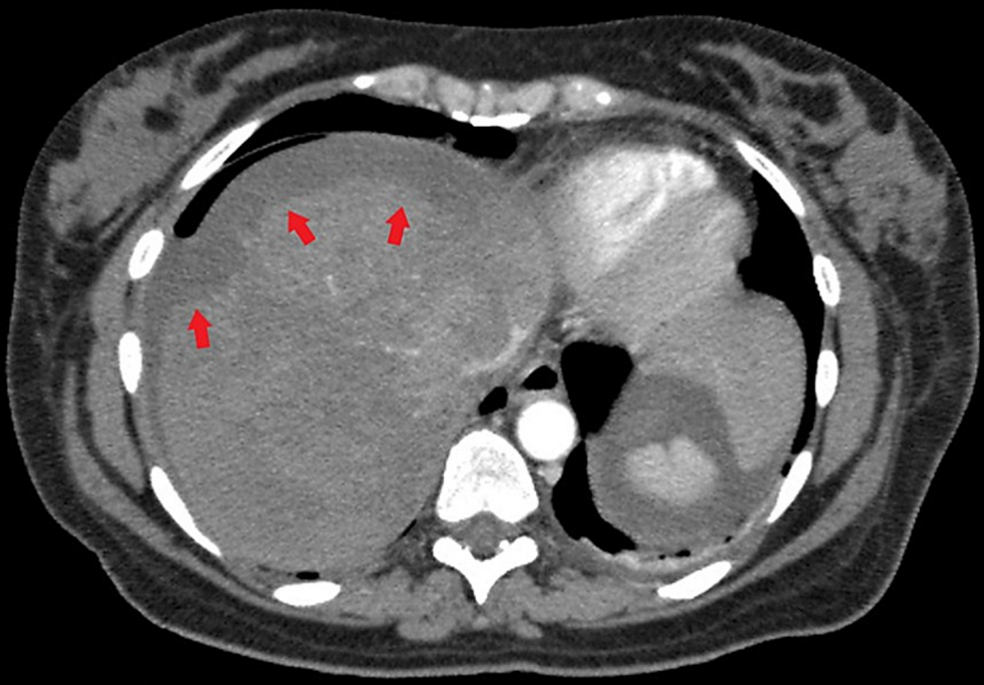
Abdominal CT-Scan in the transverse plane showing a large subcapsular liver infiltrate consistent with a hematoma. The capsule is intact.

An emergency cesarean section was performed, due to the severe worsening of the patient’s clinical condition and elevation in blood pressure, by Pfannenstiel incision. A 2,490-g live-born male infant was delivered and the patient was then transferred to the ICU for further monitoring of the blood pressure. Due to the presence of hypogastric tenderness and elevated liver function tests (LFT), abdominal ultrasonography was performed. Transabdominal USG (TA-USG) delineated a hyperechoic heterogenous crescentic mass, measuring 6.3 × 2.3 cm in diameter on the diaphragmatic surface of the right hepatic lobe, involving segments V-VI, in addition to a small quantity of free fluid in the Douglas and Morrison pouch allowing for the diagnosis of subcapsular liver hematoma to be made. Abdominal computed tomography (CT) depicted the hematoma had covered segments VIII, VII, V, and VI (Figure 1). Additionally, free hemorrhagic fluid with a density of 30 Hounsfield units (HU) was localized perihepatic and perisplenic between the intestinal loops, mainly extending from the paracolic area to the lesser pelvis. The patient had persistent tachycardia but no hypotension. In the first 24 hours following delivery, the patient was immediately transfused and a Hepatopancreatobiliary consultation was obtained. Based on the response to the transfusions she was transferred to the ICU of a Tertiary Hospital for further monitoring and management. She immediately underwent a liver angiogram that did not show any pseudoaneurysms or sites of extravasation in the liver. Overall, hemoglobin level dropped 3.5 units, from 12.7 g/dL to 9.2 g/dL, 24 hours after the cesarean section. The patient required several transfusions of packed red cells and fresh frozen plasma over the following days and her hemodynamic profile gradually improved. Eventually, she was transferred to the regular ward and was discharged several days later. During her stay in the maternity hospital, she received a total of 10 units of packed red blood cells along with 12 units of fresh frozen plasma. Presently, she is completely asymptomatic while the follow-up CT scan showed gradual absorption of the liver hematoma.

## Discussion

SLH can present as spontaneous bleeding from complications with HELLP syndrome due to severe pre-eclampsia. It occurs between the Glisson’s capsule and the liver parenchyma and can significantly increase the rate of maternal mortality, ranging between 17% and 59%, and its consequences are further dependent on accurate timing of diagnosis and availability of therapeutic interventions [2]. The incidence of SLH is reported to be found in 2% of pregnancies complicated by HELLP syndrome with an increased risk in women with advanced age and/or multiparity (10 times higher incidence than primiparous women) [3,4]. Not sufficient data exist concerning the ethnic background of the patients with SLH, but we do know that HELLP syndrome has a higher incidence in White women when compared with Black women (30 vs 19; [P=0.03] in a study performed among 473 pregnancies) [5]. Spontaneous rupture of the liver in pregnant women was first described in 1844 by Abercrombie [6], where since then, 35 cases have been reported in the international literature until 2017 [7].

The pathophysiology behind end-organ damage in HELLP syndrome is not fully elucidated. Ospina-Tascón et al., in a study of the microcirculatory dysfunction in severe pre-eclampsia in women with HELLP syndrome, found that the combination of microangiopathy and vasospasm are responsible for the end-organ complications [8]. In SLH during pregnancy, as Aziz et al. observed with the help of immunofluorescence studies, fibrin is deposited in the hepatic sinusoids and this event is considered to be the first one among a cascade of platelet activation and aggregation into thrombus, with subsequent occlusion of the capillaries and hemorrhagic necrosis of the liver [3]. Histopathologically, the SLH is described as periportal or focal parenchymal necrosis caused by microaneurysms [4]. Thus, the pre-existing endothelial dysfunction and intravascular hypovolemia secondary to preeclampsia in combination with the coagulopathy, the altered histology of the liver, and the increased arterial blood pressure leads to a transformation of the liver necrosis into intrahepatic hemorrhage, that expands until a subcapsular hematoma is formed [9]. Minor trauma such as abdominal palpation, uterine contractions or vomiting may lead to a continued expansion of the hematoma and rupture of the hepatic capsule [4], an event that fortunately was not observed in this patient presentation.

The clinical and laboratory presentation of SLH can be nonspecific. These may include; hemodynamic instability and sudden onset pain in the right upper quadrant, epigastric, severe pain in the shoulder, and/or nausea and vomiting due to distension of the hepatic parenchyma and Glisson’s capsule [2]. In the designated case, the patient did not present an elevation of LFT, but rather, elevated BP and uric acid, in addition to abdominal pain with right shoulder radiation and vomiting. Care must be taken with such patient presentation, as to not misdiagnose it as another intra-abdominal pathology or even pulmonary embolism [2]. Known patient history of preeclampsia should help in guidance toward a prompt diagnosis of potential complications, such as HELLP, and the sequelae which may present [10]. In the laboratory, in addition to the sudden drop in hematocrit, there is a large increase in transaminases (>1,000 IU/L), a drop in platelets and disturbances of coagulation factors. The use of paraclinical imaging studies should be chosen based on availability and ease of use. Ultrasound is both portable and can give prompt confirmation in suspected diagnosis. Whereas CT can provide a proper classification and staging in addition to information regarding hematoma extend and proper visualization of other surrounding anatomical structures and their potential involvement. Furthermore, serial imaging investigations can be performed in order to visualize the size of the SLH and if the expertise allows, percutaneous embolization of the hepatic arteries may be attempted [4].

For proper classification of liver injury and guidance for proper treatment, the American Association for the Surgery of Trauma (AAST) is used to grade the extent of hepatic trauma between grades I and VI (Table 2). There are no specified guidelines used in order to aid in the clinical decision-making process, but AAST is useful in providing some direction in how to ensure adequate patient care [11].

**Table 2:** Liver injury classification by the American Association for the Surgery of Trauma. Adapted from [12].

Grade	Type of Injury	Description of Injury
I	Hematoma Laceration	Subcapsular, <10% surface area Capsular tear, <1cm parenchymal depth
II	Hematoma Laceration	Subcapsular, 10%-50% surface area Intraparenchymal <10 cm diameter Capsular tear 1-3 cm parenchymal depth, <10 cm length
III	Hematoma Laceration	Subcapsular, >50% surface area of ruptured subcapsular or parenchymal hematoma Intraparenchymal >10 cm Capsular tear >3 cm parenchymal depth
IV	Laceration	Parenchymal disruption involving 25%-75% hepatic lobe or involves 1-3 Couinaud segments
V	Laceration Vascular	Parenchymal disruption involving >75% of hepatic lobe Juxtahepatic venous injuries (retrohepatic vena cava / central major hepatic veins)
VI	Vascular	Hepatic Avulsion

Approaches to treatment can be conservative or invasive and further guided by the staging via AAST. In hemodynamically stable patients, conservative management is successful with the administration of transfused fluids and blood products in order to correct the coagulopathy. In addition, serial imaging investigations can be performed in order to visualize the size of the SLH [13]. Conversely, surgical management is indicated for patients with poor evolution in addition to blood transfusions. Such procedures can include perihepatic packing and drainage of the surgical site, ligation of the appropriate branch of the portal vein or hepatic artery, patching of the omentum, and partial liver resection. Liver transplantation has been performed when hemorrhaging was unable to be contained [13]. Although the patient, in this case, was graded class IV with AAST, she responded well to conservative management with fluid, blood transfusions, and continuous monitoring. Beginning by following hematocrit hourly followed by subsequence imaging investigations performed daily to observe the evolution of the hematoma.

## Conclusions

The purpose of this case report and brief literature review is to raise awareness of such a rare clinical complication, emphasizing that early detection can prove life-saving for both the mother and the newborn. Hepatic rupture or hematoma is a factor that should be considered in hypertensive pregnant women who also present epigastric and/or right hypochondrial pain, pallor, and hypotension before or after delivery. Ultrasound can be used for the diagnosis of this complication, although a CT scan is more sensitive. MRI is reserved for diagnosis in pregnant women or non-bleeding situations. Intense in-hospital monitoring of vital signs, paraclinical investigations, and a diligent multidisciplinary team are the cornerstones for the correct management of these patients. The first line of treatment in a hemodynamically stable patient is a conservative one, as demonstrated in this report, successfully increasing the likelihood of survival. Conservative management, including intensive medical support with infused fluid, and replacement of blood and blood products could be applied in non-bleeding situations and for patients with stable hemodynamics. In conclusion, this case report demonstrates that a pregnant woman with SLH as a complication of HELLP syndrome can be successfully managed non-surgically if her hemodynamic status is stable and is closely monitored in a tertiary center.
